# Spontaneous Rupture of Splenic Artery Aneurysm

**DOI:** 10.7759/cureus.50937

**Published:** 2023-12-22

**Authors:** Márcia Carvalho, João Mendes, Juliana Pereira-Macedo, Margarida Vinagreiro, Ricardo Lemos

**Affiliations:** 1 Surgery, Centro Hospitalar do Médio Ave, Santo Tirso, PRT

**Keywords:** treatment, splenectomy, rupture, aneurysm, splenic artery

## Abstract

Splenic artery aneurysms are rare and usually asymptomatic, with a high risk of mortality once they get ruptured. A case report of a spontaneous rupture of a splenic artery aneurysm in a 65-year-old female is reported.

The patient presented in the emergency department with abdominal pain, nausea, and vomiting, followed by syncope. A contrast-enhanced CT scan was performed and showed a splenic artery aneurysm measuring 40 × 35 mm surrounded by a hematoma. The patient was submitted to emergency laparotomy with ligation of the splenic artery, aneurysm resection, and splenectomy. There were no surgical complications, and the patient was discharged home on the fifth postoperative day.

A rupture of a splenic aneurysm is a rare condition with a high mortality rate and should be considered a differential diagnosis in a patient with abdominal pain and hemodynamic instability.

## Introduction

Splenic artery aneurysm is a rare condition with an estimated prevalence of 1% in the general population [1,2]. However, it is the third most common arterial aneurysm, only exceeded by aneurysms in the abdominal aorta and iliac arteries. Described diameters range from 0.6 to 30 cm [3], and it is four times more common in females [4].

﻿Although the pathogenesis is not fully understood, risk factors include fibromuscular dysplasia, collagen vascular diseases, female gender, history of multiple pregnancies, and portal hypertension [2,4]. As elsewhere, true aneurysms involve the three layers of the arterial wall, while pseudoaneurysms lack one or more of the arterial layers, which explains the higher risk for rupture [4]. True aneurysms are most commonly located in the distal third of the splenic artery (75%), followed by the middle third in 20% [5].

Patients typically present in the sixth or seventh decade of life with nonspecific abdominal pain, although splenic artery aneurysms can also be asymptomatic [4]. When ruptured, they can have a catastrophic presentation with ﻿intraperitoneal hemorrhage and hypovolemic shock, which has a high mortality rate ﻿(25%-75%) [2,3].

## Case presentation

A 65-year-old female with no relevant past medical history presented to the emergency department in a medium-sized hospital in Portugal with a previous episode of nausea and vomiting, diffuse abdominal pain, and syncope starting the day before.

**Figure 1:**
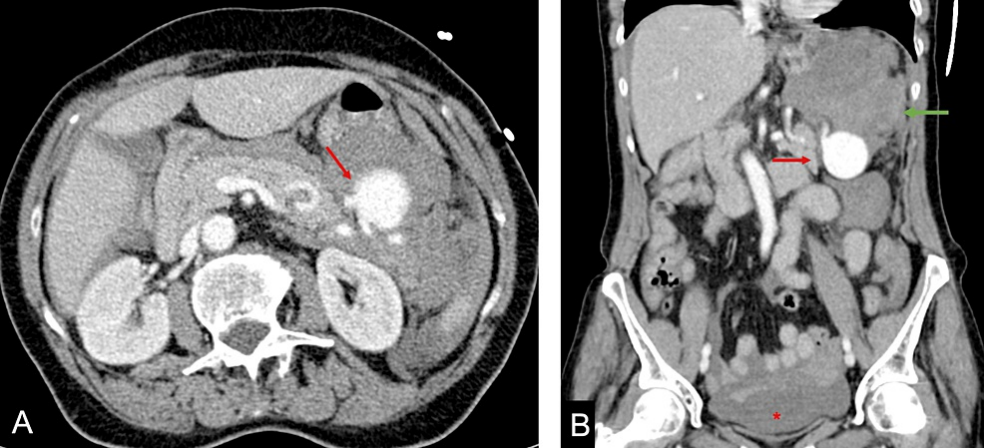
Contrast-enhanced CT axial (A) and coronal (B) reconstruction showing a saccular splenic artery aneurysm (red arrows), with adjacent hematoma (green arrow) and hemoperitoneum (asterisk).

**Figure 2:**
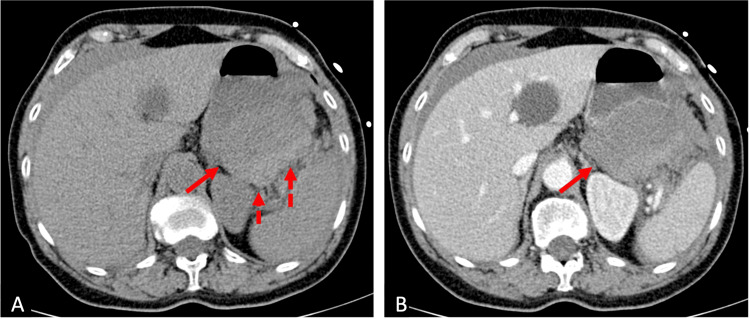
Non-contrast-enhanced (A) and contrast-enhanced (B) CT scan showing a voluminous hematoma (arrows) between the pancreatic tail and posterior stomach wall, with spontaneously hyperdense content in a dependent position (dashed arrows) indicating fresh blood.

On admission, the patient had another syncope and presented with signs of shock: confused, pale, tachycardic (heart rate of 110 beats/minute), and hypotensive (blood pressure of 70/40 mmHg). Abdominal examination showed no signs of peritoneal irritation. The laboratory workup showed anemia (8.0 g/dL {reference: 12.0-16.0 g/dL}), leukocytosis (17.65 × 10^3^/uL {reference: 4.0-11.0 x 10^3^/uL}), and elevated C-reactive protein (13.00 mg/dL {reference: 0-0.5 mg/dL}). Arterial blood gas evaluation demonstrated metabolic acidosis and elevated lactates (10 mmol/L {reference: 0.5-1.5 mmol/L}). Abdominal CT revealed significant hemoperitoneum in the left upper quadrant, between the splenic hilum and the pancreatic tail, associated with a partially thrombosed splenic artery aneurysm measuring 4 × 3.5 cm (Figures 1, 2).

**Figure 3:**
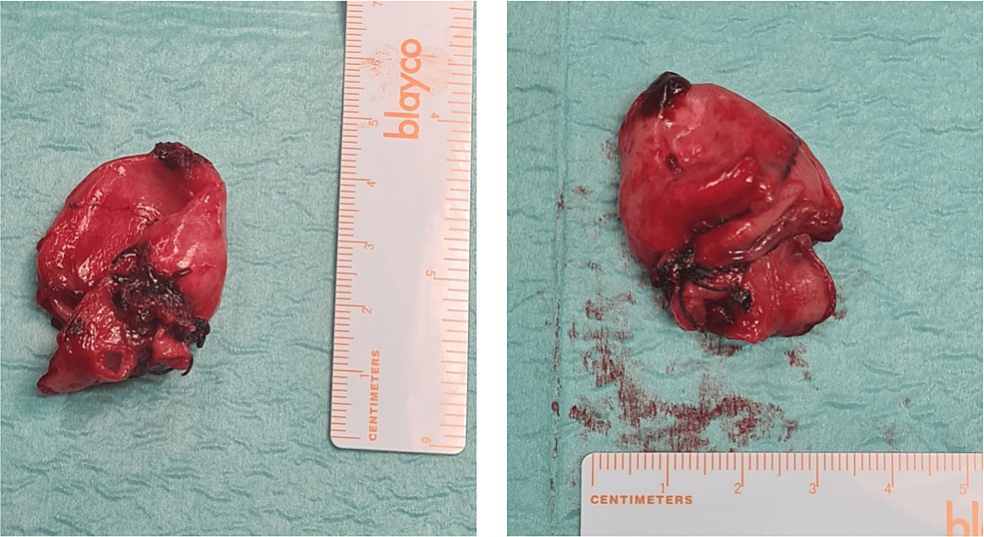
Saccular splenic aneurysm after resection.

**Figure 4:**
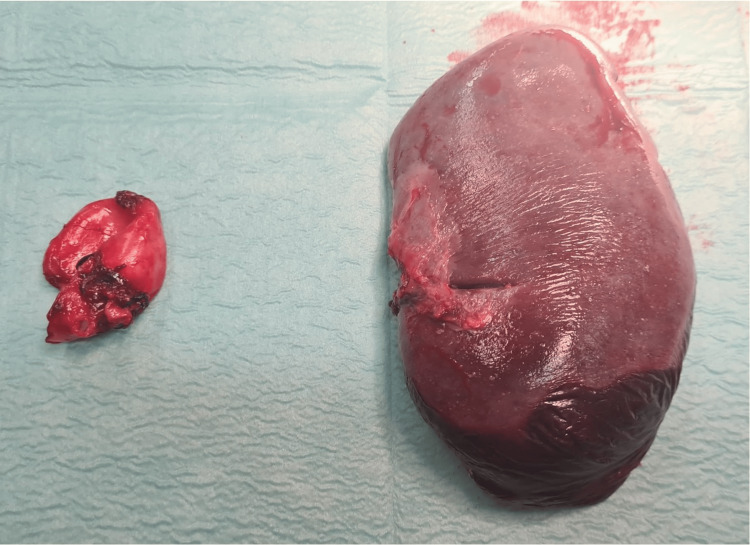
Saccular splenic aneurysm and the spleen after resection.

The patient went immediately to the operating room and underwent exploratory laparotomy, where voluminous hemoperitoneum was found. A ruptured saccular aneurysm in the mid-third of the splenic artery was identified as the source of bleeding. The splenic artery was ligated proximally, with resection of the aneurysm, followed by splenectomy (Figures 3, 4). During surgery, there was an estimated blood loss of 3 L, resulting in hypovolemic shock and the need for a massive transfusion with four units of plasma, four of platelets, and four of red blood cells.

The patient was transferred to the ICU for overall support, where she remained for two days. The patient was medicated with intravenous (IV) paracetamol 1000 mg and tramadol 50 mg three times a day, IV omeprazole 40 mg/day, IV metoclopramide 10 mg three times a day, and a subcutaneous injection of enoxaparin 40 mg/day. Afterward, she was moved to the general ward for three days and was discharged home on the fifth postoperative day. There were no surgical complications. The diagnosis of ruptured splenic artery aneurysm was confirmed by pathology. Postsplenectomy vaccination protocol was performed two weeks after the surgical procedure.

## Discussion

Splenic artery aneurysms were first described in 1770 by Beaussier in autopsies. More than a hundred years passed before the first preoperative diagnosis was made, in 1920 by Hoegler, and it was only in 1940 that the first surgical intervention was described [4,6]. Around 80% of splenic artery aneurysms are asymptomatic and diagnosed incidentally during imaging studies [3]. When symptoms are present, they are usually nonspecific and variable but most commonly include epigastric and left upper quadrant abdominal pain [6,7].

Aneurysmal rupture has been reported in 3%-10% of all splenic aneurysms, and it can cause a dramatic hypotensive shock with a high mortality rate [6]. The risk of rupture is higher in larger aneurysms, with a cutoff of 2 cm in diameter used as a high-risk feature [2]. Additional risk factors include pregnancy, cirrhosis, and liver transplantation [8].

A rare presentation occurs with the double-rupture phenomenon. The first hemorrhage is contained by the lesser omental sac, leading to temporary tamponade, and the patient remains stable with minimal clinical symptoms. After 6-96 hours, the blood overflows into the peritoneal cavity through the foramen of Winslow with resultant severe shock [4].

Elective treatment is indicated for all symptomatic splenic artery aneurysms, regardless of their dimensions [7]. Furthermore, treatment is also recommended for lesions ≥2 cm, growing aneurysms, all pregnant or fertile females, patients with portal hypertension, or candidates for liver transplantation [9]. Elective treatment depends on the aneurysm's location, patient's age, operative risks, and clinical status [8].

The endovascular procedure is considered the first choice of treatment for splenic artery aneurysm. When the patient is unstable or endovascular procedures are not available, open surgery is the best approach [9]. The surgical management of a ruptured splenic artery aneurysm consists of aneurysm resection, with or without splenectomy [8]. Although splenic preservation is desirable, it is difficult to achieve in an emergency setting with a ruptured splenic aneurysm [3].

This is a rare case in the literature of a splenic artery aneurysm that ruptured spontaneously in a healthy patient with no risk factors. Immediate diagnosis, rapid resuscitation, and surgical intervention were essential for the patient's survival.

Most cases described in the literature are reported in pregnant, postpartum females or associated with risk factors for rupture.

However, there are few cases in the literature of splenic artery aneurysm rupture in patients without significant risk factors or identifiable causes. As in our patient, these cases often challenge understanding the exact mechanism or trigger for the rupture, as the patient may not have the typical predisposing conditions commonly associated with this complication.

Our patient is a healthy patient, with no trauma history, pregnancy, or other known risk factors.

The lack of identifiable risk factors in certain cases highlights the complexity of this condition and the need for continued research to understand better the underlying mechanisms leading to a spontaneous rupture in seemingly low-risk patients.

## Conclusions

This is a rare case of a spontaneous splenic aneurysm rupture in a previously healthy female without relevant risk factors. The patient was admitted to the emergency department with signs of shock, and a prompt diagnosis, resuscitation, and surgical treatment were crucial to save the patient's life. In the literature, there are few cases described of splenic aneurysm rupture, but the majority of cases described are in pregnant females.

Although splenic aneurysm rupture is a rare cause of hypovolemic shock in the emergency department, the diagnosis should be considered in patients with abdominal pain and signs of hypovolemia so that a timely and appropriate lifesaving intervention can be performed, which requires an aggressive surgical approach.

## References

[1] Abdulrahman A, Shabkah A, Hassanain M, Aljiffry M (2014). Ruptured spontaneous splenic artery aneurysm: a case report and review of the literature. Int J Surg Case Rep.

[2] Anand A, Khurana S, Ateriya N, Sunil Kumar Sharma GA (2022). Sudden death due to non-traumatic rupture of splenic artery aneurysm. Med Leg J.

[3] Hosseinzadeh A, Shahriarirad R, Asgharzadeh Majdazar V, Moeini Farsani M, Tadayon SM (2022). Spontaneous rupture of a large splenic artery aneurysm in a 59-year-old male patient with pemphigus vulgaris: a case report. J Med Case Rep.

[4] Al-Habbal Y, Christophi C, Muralidharan V (2010). Aneurysms of the splenic artery - a review. Surgeon.

[5] Berceli SA (2005). Hepatic and splenic artery aneurysms. Semin Vasc Surg.

[6] Pararas N, Rajendiran S, Taha I, Powar RR, Holguera C, Tadros E (2020). Spontaneous rupture of a huge splenic artery aneurysm: a case report. Am J Case Rep.

[7] Akbulut S, Otan E (2015). Management of giant splenic artery aneurysm: comprehensive literature review. Medicine (Baltimore).

[8] Abbas MA, Stone WM, Fowl RJ (2002). Splenic artery aneurysms: two decades experience at Mayo Clinic. Ann Vasc Surg.

[9] Ali S, Verma V, R S, Wani I (2011). Giant splenic artery aneurysm: case report. ISRN Surg.

